# Comparison of Nitrogen Oxide Metabolism among Diverse Ammonia-Oxidizing Bacteria

**DOI:** 10.3389/fmicb.2016.01090

**Published:** 2016-07-12

**Authors:** Jessica A. Kozlowski, K. Dimitri Kits, Lisa Y. Stein

**Affiliations:** Department of Biological Sciences, Biological Sciences Building, University of Alberta, Edmonton, ABCanada

**Keywords:** nitrogen oxides, nitrifier denitrification, ammonia-oxidizers, *Nitrosomonas*, *Nitrosospira*, nitrous oxide, nitric oxide, chemodenitrification

## Abstract

Ammonia-oxidizing bacteria (AOB) have well characterized genes that encode and express nitrite reductases (NIR) and nitric oxide reductases (NOR). However, the connection between presence or absence of these and other genes for nitrogen transformations with the physiological production of nitric oxide (NO) and nitrous oxide (N_2_O) has not been tested across AOB isolated from various trophic states, with diverse phylogeny, and with closed genomes. It is therefore unclear if genomic content for nitrogen oxide metabolism is predictive of net N_2_O production. Instantaneous microrespirometry experiments were utilized to measure NO and N_2_O emitted by AOB during active oxidation of ammonia (NH_3_) or hydroxylamine (NH_2_OH) and through a period of anoxia. This data was used in concert with genomic content and phylogeny to assess whether taxonomic factors were predictive of nitrogen oxide metabolism. Results showed that two oligotrophic AOB strains lacking annotated NOR-encoding genes released large quantities of NO and produced N_2_O abiologically at the onset of anoxia following NH_3_-oxidation. Furthermore, high concentrations of N_2_O were measured during active O_2_-dependent NH_2_OH oxidation by the two oligotrophic AOB in contrast to non-oligotrophic strains that only produced N_2_O at the onset of anoxia. Therefore, complete nitrifier denitrification did not occur in the two oligotrophic strains, but did occur in meso- and eutrophic strains, even in *Nitrosomonas communis* Nm2 that lacks an annotated NIR-encoding gene. Regardless of mechanism, all AOB strains produced measureable N_2_O under tested conditions. This work further confirms that AOB require NOR activity to enzymatically reduce NO to N_2_O in the nitrifier denitrification pathway, and also that abiotic reactions play an important role in N_2_O formation, in oligotrophic AOB lacking NOR activity.

## Introduction

Chemolithotrophic ammonia-oxidizing bacteria (AOB) are important players in the global biogeochemical nitrogen cycle and perform the first step in nitrification; the oxidation of ammonia (NH_3_) to nitrite (${\mathrm{NO}}_{2}^{-}$). AOB are abundant in a vast array of environments including soils, marine and fresh-water, and wastewater treatment plants (Klotz et al., 2006; Norton et al., 2008; Jia and Conrad, 2009; Ke et al., 2015) and are implicated in production of nitrous oxide (N_2_O) through enzymatic (Stein, 2011; Kozlowski et al., 2014) and abiotic processes (Jones et al., 2015; Zhu-Barker et al., 2015). AOB have the potential to utilize ${\mathrm{NO}}_{2}^{-}$ as an alternate terminal electron acceptor through the process of nitrifier denitrification (Stein, 2011) resulting in net production of N_2_O (Stein and Yung, 2003; Kool et al., 2011; Zhu et al., 2013). N_2_O has been measured from pure cultures of AOB from both the *Nitrosomonas* (Poth and Focht, 1985; Kozlowski et al., 2014) and *Nitrosospira* (Dundee and Hopkins, 2001; Wrage et al., 2004; Shaw et al., 2006) genera. However, studies on the enzymology and pathways of N_2_O production by AOB have mostly focused on *N. europaea* ATCC 19718 (Beaumont et al., 2002, 2004; Cantera and Stein, 2007; Yu and Chandran, 2010; Yu et al., 2010; Kozlowski et al., 2014) leaving open the possibility that not all AOB strains share equivalent pathways and regulatory mechanisms.

The nitrifier denitrification pathway includes a nitrite reductase (NIR) to reduce ${\mathrm{NO}}_{2}^{-}$ to nitric oxide (NO) and nitric oxide reductase (NOR) to reduce NO to N_2_O. All closed AOB genomes, with the exception of *N. communis* Nm2 (Kozlowski et al., 2016b), have genes encoding the copper-containing NirK (Prosser et al., 2014). Furthermore, all AOB encode NOR genes (*norB* and/or *norY*) with the exception of *Nitrosomonas* sp. Is79A3 (Bollmann et al., 2013) and *N. ureae* Nm10 (Kozlowski et al., 2016a). Both *Nitrosomonas* sp. Is79A3 and *N. ureae* Nm10 are considered oligotrophic, growing optimally in medium containing 1–5 mM ammonium (Prosser et al., 2014). In contrast, *N. communis* Nm2 is considered eutrophic and prefers higher concentrations of 10–50 mM ammonium (Prosser et al., 2014).

Previous studies on the model organism *N. europaea*, a eutrophic strain, showed that both hydroxylamine (NH_2_OH) oxidation and ${\mathrm{NO}}_{2}^{-}$ reduction can lead to significant emission of N_2_O (Cantera and Stein, 2007; Kozlowski et al., 2014). Previous work also revealed that NorB, but not NirK, is required for production of N_2_O by *N. europaea* (Kozlowski et al., 2014). This observation, in addition to the lack of annotated NIR or NOR genes in some closed AOB genomes, has brought into question whether all AOB can even perform nitrifier denitrification and emit N_2_O under similar conditions as *N. europaea*. There is also a question of whether uncharacterized NIR and/or NOR enzymes are expressed in AOB that can contribute to the process. The production and metabolism of NO and its role in N_2_O emission is another understudied aspect of nitrogen oxide metabolism in AOB; *N. multiformis* ATCC 25196 was recently found to emit large quantities of NO during active NH_3_-oxidation (Kozlowski et al., 2016c).

Due to the lack of comparative information on nitrogen oxide metabolism in AOB, five strains representing different phylogenies and trophic states and with closed genomes were selected for this study. Our main objectives were to: (i) compare NO and N_2_O production profiles of the five strains during NH_3_ and NH_2_OH oxidation and over a period of anoxia when nitrifier denitrification is most active in *N. europaea*, and (ii) determine whether gene content, trophic state, and/or phylogeny of these diverse AOB were predictive of their capacity to metabolize and/or emit NO or N_2_O.

## Materials and Methods

### Strains and Cultivation

AOB strains included *N. europaea* ATCC 19718^T^, *N. communis* strain Nm2^T^, *Nitrosomonas* sp. Is79A3, *N. ureae* Nm10^T^, and *N. multiformis* ATCC 25196^T^. All strains have closed genomes and grow under similar cultivation conditions to allow for proper comparisons across phylotypes and trophic status. Furthermore, an AOB strain was selected from each cluster in the *Betaproteobacteria* with a cultured representative, 3, 6, 7, and 8 (based on 16S rRNA phylogeny; Norton, 2011), with the exception of the newly cultured cluster 0 *N. lacus* sp. nov. as its genome is not yet closed (Garcia et al., 2013; Urakawa et al., 2014). AOB cultures were grown and maintained in Wheaton bottles (250 mL) sealed with caps inlayed with butyl rubber stoppers at 28°C in 100 mL HEPES-buffered HK medium (Krümmel and Harms, 1982) and phenol red as pH indicator (pH of 7.5–8) with either 5 mM (NH_4_)_2_SO_4_ for the meso- and eutrophic strains (*N. europaea*, *N. communis*, and *N. multiformis*), or 2.5 mM (NH_4_)_2_SO_4_ for the oligotrophic strains (*Nitrosomonas* sp. Is79A3 and *N. ureae*; Prosser et al., 2014). All cultures were transferred (5% v/v inoculum) when ca. 80% of the NH_3_ substrate was consumed as determined by ${\mathrm{NO}}_{2}^{-}$ concentration (Bollmann et al., 2011). The pH of all cultures was adjusted as needed with 10% NaHCO_3_.

### Phylogenetic and Genome Analysis of AOB

PhyloPhlAn (Segata et al., 2013) was used to generate and analyze the genome-wide phylogeny of AOB. Genomes of 14 AOB were obtained from the National Center for Biotechnology Information^1^. All of the predicted protein-coding sequences for each genome were exported into PhyloPhlAn to identify and align 400 broadly conserved protein sequences between all of the input genomes. PhyML 3.0 (Guindon et al., 2010) was used to construct a maximum likelihood phylogeny using the *Gammaproteobacteria* as the root and node support was calculated using 500 bootstrap replicates.

### Microrespirometry Experiments

Instantaneous microrespirometry (MR) experiments of AOB are described in detail elsewhere (Kozlowski et al., 2016c). Briefly, MR experiments were performed at 28°C in a 10 mL 2-port injection lid glass chamber (Unisense, Aarhus, Denmark). For instantaneous experiments all strains were grown to late-log phase (7–8 mM ${\mathrm{NO}}_{2}^{-}$), filtered on Supor^®^ 200 0.2 μm filters (Pall, Ann Arbor WI), and rinsed three times with NH_3_-free HK media (Krümmel and Harms, 1982). Ca. 1 × 10^10^ total cells were used per experiment for all strains as determined by direct cell count by phase-contrast light microscopy. All cells for instantaneous MR measurements were in a planktonic state, re-suspended in NH_3_-free HK medium and provided either 2 mM NH_4_Cl as substrate or pulses of 250 μM or 100 μM NH_2_OH-HCl (final chamber concentration; 99.999% purity, Sigma–Aldrich, St Louis, MO, USA). Previous testing revealed that all strains could tolerate up to 250 μM NH_2_OH (final chamber concentration) with the exception of *N. communis* which was unable to tolerate more than 100 μM NH_2_OH (final chamber concentration) per injection (data not shown). Chamber O_2_ was determined by an O_2_ electrode (OX-MR 500 μm tip diameter MR oxygen electrode; Unisense, Aarhus, DenmarK), N_2_O concentration was measured using an N_2_O-500 N_2_O minisensor electrode with 500 μm tip diameter (Unisense, Aarhus, DenmarK), and NO was measured using an ami-600 NO sensor with 600 μm tip diameter (Innovative Instruments Inc., Tampa, FL, USA). The availability of O_2_ in the MR chamber, a closed system, was ca. 243 μM O_2_ based on equilibrium O_2_ concentration at operating temperatures and medium salinities for experiments performed without N_2_-sparged medium.

### Chemical Controls

Chemical controls were performed to determine the production of N_2_O from reactivity of NH_2_OH with media + ${\mathrm{NO}}_{2}^{-}$, or from killed-cells (1 × 10^10^ total cells) with media + NH_2_OH. Chemical controls used N_2_-sparged medium (to achieve 0–3% O_2_ saturation in liquid phase) containing 250 μM NaNO_2_ and then adding 250 μM NH_2_OH (final chamber concentration) to reflect conditions in the chamber when testing for ${\mathrm{NO}}_{2}^{-}$ consumption by AOB as an alternate terminal electron acceptor with NH_2_OH as the electron donor. Cells for control experiments were heat-killed by boiling for 30 min. The heat-killed cell controls involved addition of 250 μM NH_2_OH to the MR-chamber containing N_2_-sparged media with 1 × 10^10^ total heat-killed cells of each AOB strain. N_2_O was measured as described above.

## Results and Discussion

### Phylogeny and Comparative Gene Inventory of AOB

**FIGURE 1:**
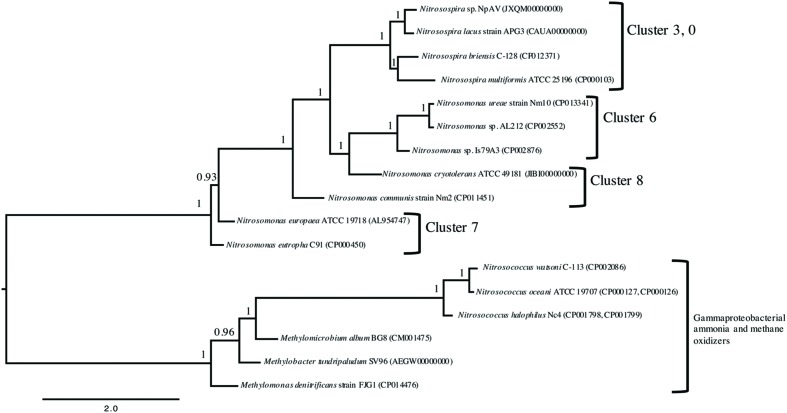
**Rooted maximum likelihood phylogeny of 14 publically available genomes of ammonia oxidizing bacteria based on 400 broadly conserved amino-acid sequences.** PhyloPhlAn (Segata et al., 2013) was used to identify and align the amino-acid sequences in all input genomes. The tree was constructed using PhyML 3.0 (Guindon et al., 2010) with the *Gammaproteobacteria* as the root. Bootstrap values (as a proportion of 500 replicates) are denoted above the branches and branch lengths correspond to sequence differences as indicated by the scale bar at the bottom.

A whole-genome analysis utilizing PhyloPhlAn showed that each of the 5 *Betaproteobacteria* AOB chosen for physiological analysis in the present study separated into individual clades (**Figure 1**). The separation of each AOB into a unique branch, using 400 core protein markers from available complete genome sequences to form a high-resolution tree, shows a clearer and greater separation than currently available 16S rRNA or *amoA* single gene sequence phylogenies (Norton, 2011). The results of this multiple-marker, genome-wide, comparison highlight a need to reevaluate and perhaps reclassify some members of *Nitrosomonas* into different genera.

Comparison of inventory involved in central ammonia-oxidizing metabolism and NOx production revealed differences across the 5 strains (**Table 1**). In agreement with previous analyses of AMO gene clusters in betaproteobacterial AOB (Klotz and Stein, 2011) all AOB of the current study contain 1–2 copies of the *amoCABED* cluster encoding ammonia-monooxygenase (**Table 1**). All strains encoded at least one monocistronic copy of the *amoC* gene with the exception of *N. communis* (**Table 1**), a feature shared in common with the gammaproteobacerial AOB (Klotz et al., 2006; Arp et al., 2007; Campbell et al., 2011). The singleton AmoC is proposed to participate in cellular recovery from stressors such as elevated temperatures and starvation by stabilizing the AMO complex in the membrane of *N. europaea* (Berube and Stahl, 2012). Also, every strain encoded at least one copy of the *amoD* gene in tandem with *amoE*, a common feature of betaproteobacterial AOB still needing biochemical characterization (Klotz and Stein, 2011). It is also common for betaproteobacterial AOB to encode 2–3 complete or incomplete (lacking *cycB*) copies of the *haoAB-cycAB* cluster (Arp et al., 2007). However, *N. ureae* represents the first sequenced AOB to harbor 4 complete copies of the Hydroxylamine dehydrogenase (HAO) gene cluster (**Table 1**). Knockouts of one or two *haoA* gene copies from *N. europaea* did not result in a significant phenotype (Hommes et al., 2002), suggesting that the multiple copies are isofunctional. However, knockouts of individual *amoA* or *amoB* gene copies in *N. europaea* did result in different phenotypes, suggesting that operons encoding AMO are differentially regulated (Stein et al., 2000). For *N. ureae* and perhaps *Nitrosomonas* sp. AL212 (Suwa et al., 2011), additional gene clusters encoding AMO and HAO could be a strategy to thrive in oligotrophic environments to gain maximum reductant from available substrate; however, further studies are required to validate whether all of the gene copies are expressed, isofunctional, and/or differentially regulated. As with *N. europaea* and *N. eutropha*, one copy of the HAO gene cluster in *N. communis* lacks *cycB* (**Table 1**), encoding cytochrome C_m_552 (Arp et al., 2007). All strains, with the exception of *Nitrosomonas* sp. Is79A3 (Bollmann et al., 2013), encode the AOB-specific red copper protein nitrosocyanin (**Table 1**) proposed to be involved in the NH_3_-oxidation pathway as a redox sensitive electron carrier (Arciero et al., 2002; Sayavedra-Soto and Arp, 2011).

**Table 1:** Annotated gene inventory with implications in ammonia-oxidation or N-oxide metabolism from complete genomes of *Betaproteobacteria* AOB utilized in the present study.

Strain	*Nitrosomonas europaea* ATCC 19718	*Nitrosomonas communis* Nm2	*Nitrosomonas* sp. Is79A3	*Nitrosomonas ureae* Nm10	*Nitrosospira multiformis* ATCC25196
Ammonia monooxygenase (*AMO*)	*amoCABED* NE2064-59 NE0945-40 *amoC* NE1411	*amoCABED* AWW31_01090-70 AWW31_05385-65	*amoCABED* Nit79A3_0471-75 Nit79A3_2886-82 *amoCAB*Nit79A3_1079-81 *amoC* Nit79A3_1233 Nit79A3_1595	*amoCABED* ATY38_01315-295 ATY38_07250-70 *amoCAB* ATY38_13760-50 *amoCE* ATY38_06315-10 *amoC* ATY38_09265	*amoCABED* Nmul_A2326-22 *amoCAB* Nmul_A0798-800 *amoC* Nmul_A0177 Nmul_A2467

Hydroxylamine dehydrogenase(*HAO*)	*haoAB-cycAB* NE0962-59 NE2339-36 *haoAB-cycA* NE2044-42	*haoAB-cycAB* AAW31_01285-70 AAW31_16290-75 *haoAB-cycA* AAW31_18275-65	*haoAB-cycAB* Nit79A3_0807-10 Nit79A3_0822-25 Nit79A3_2942-39	*haoAB-cycAB* ATY38_00070-55 ATY38_06640-55 ATY38_10080-95 ATY38_15220-05	*haoAB-cycAB* Nmul_A0805-02 Nmul_A1082-85 Nmul_A2662-59

Nitrosocyanin	NE0143	AAW31_00185	Not Present	ATY38_00645	Nmul_A1601

Nitrite reductase (NirK)	*ncgABC-nirK* NE0924	Not Present	*nirK* Nit79A3_2335	*nirK* ATY38_00595	*nirK* Nmul_A1998

Cytochrome *c* nitric oxide reductases	*norCBQD* NE2003-06 *norSY-senC-orf1* NE0683-86	*norCBQD* AAW31_10555-70 *norSY-senC-orf1* AAW31_05895-910	Not Present	Not Present	*norCBQD* Nmul_A1256-43 *norSY-senC-orf1* Nmul_A2667-64

Cytochrome *c*’ beta (*cytS*)	NE0824	AAW31_17525	Nit79A3_0363	ATY38_05410	Nmul_A2484

Cytochrome P460 (*cytL*)	NE0011	AAW31_02040 AAW31_00880	Nit79A3_1628	ATY38_00655	Not Present

NO-responsive transcriptional regulator (NsrR)	NE0926	Not Present	Not Present	Not Present	Not Present

NO-responsive transcriptional regulator (NnrS)	NE1722	AAW31_04320 AAW31_06015	Nit79A3_3412	ATY38_04220	Not Present

*Locus tags from the sequenced and publicly accessible genomes are presented for each annotated gene and gene cluster.*

Analysis of NIR and NOR genes revealed that *N. communis* is the only sequenced and closed AOB genome without a copper-containing nitrite reductase (*nirK*; Kozlowski et al., 2016b) (**Table 1**). This is interesting as *nirK* is present in all published genomes of ammonia-oxidizing Thaumarchaeota (AOA; Bartossek et al., 2010), is highly expressed in metatranscriptomes (Hollibaugh et al., 2011; Radax et al., 2012), and is important for efficient substrate oxidation in *N. europaea* (Cantera and Stein, 2007; Kozlowski et al., 2014). Of the 5 strains, only *N. europaea* contains the operonic *nirK* and NO-responsive *nsrR* transcriptional regulator (Chain et al., 2003; **Table 1**), features shared by the closely related *N. eutropha* C-91 strain (Stein et al., 2007) (**Figure 1**). All the *Nitrosomonas* strains, but not *N. multiformis*, encode the NO-responsive NnrS transcriptional regulator. Two strains, *Nitrosomonas* sp. Is79A3 and *N. ureae*, both within the Cluster 6 AOB, lack annotated operons for cytochrome *c* nitric oxide reductases (Bollmann et al., 2013; Kozlowski et al., 2016a) (**Table 1**). The genome of the closely related *Nitrosomonas* sp. AL212 (**Figure 1**) does encode *norCBQD* but lacks genes for the other NOR frequently found in AOB genomes, *norSY-senC-orf1* (Suwa et al., 2011). We hypothesize that environments with low substrate availability do not experience oversaturation of NH_3_ and thus preclude accumulation of N-oxides such as NH_2_OH and NO (Hooper and Terry, 1979). Thus, NORs may not be not required by some oligotrophic AOB as nitrosative stress should be minimal. However, testing of strains such as *Nitrosomonas* AL212, an oligotrophic, NOR-encoding strain, must be accomplished to determine whether trophic state or gene content is more predictive of nitrifier denitrification activity. *N. multiformis* does not have an annotated cytochrome P460 (*cytL*) whereas *N. communis* has two copies (**Table 1**), a feature shared with *Nitrosomonas* sp. AL212 (Suwa et al., 2011). Cytochrome P460 has a proposed role in detoxification of NOx through the simultaneous oxidation NH_2_OH and NO to ${\mathrm{NO}}_{2}^{-}$ (Elmore et al., 2007; Stein, 2011) and may be important for alleviating nitrosative stress in AOB lacking NORs. All 5 genomes also contain sequences for cytochrome *c’* beta, potentially having NOR activity (Elmore et al., 2007; Stein, 2011). Future work with focus on the transcription and activities of cytochromes P460 and *c’* beta under conditions of nitrosative stress would better clarify the role of both enzymes as substitutes for lack of annotated NORs.

### Comparison of Instantaneous NOx Production from AOB during Oxidation of NH_3_ or NH_2_OH

**FIGURE 2:**
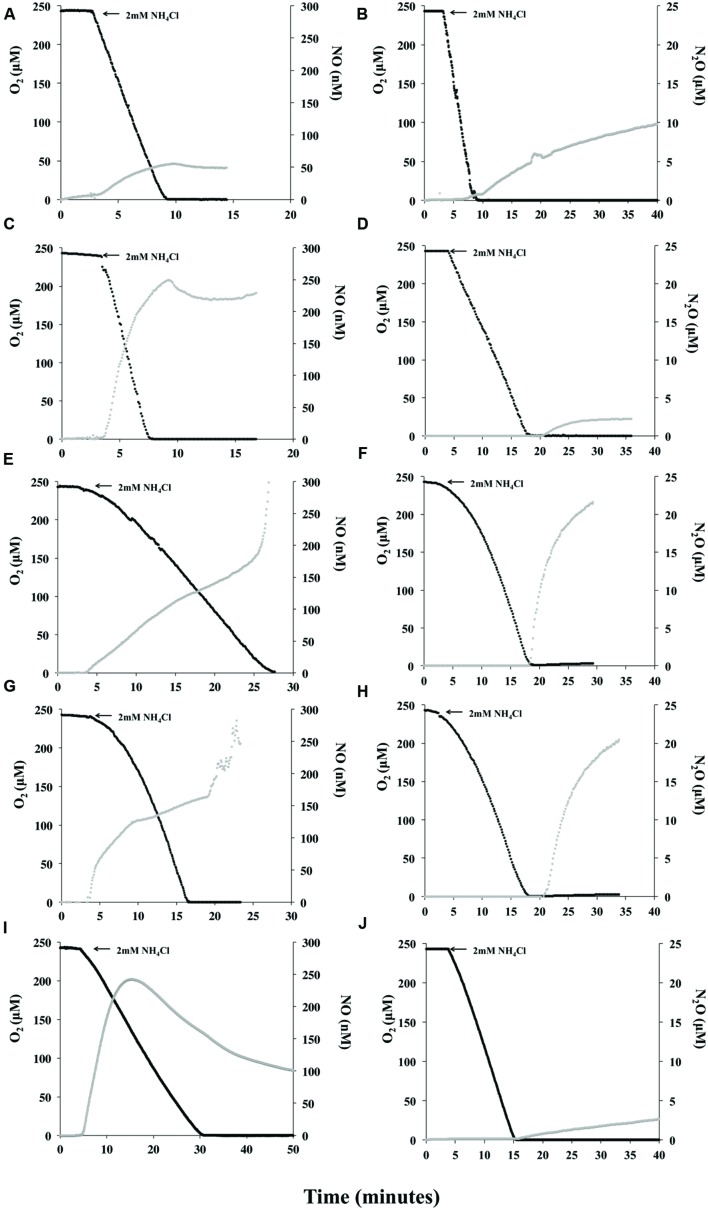
**Instantaneous measurement of O_2_ consumption and NO or N_2_O during oxidation of 2 mM NH_4_Cl.** *Nitrosomonas europaea* **(A,B)**, *N. communis* **(C,D)**, *Nitrosomonas* sp. Is79A3 **(E,F)**, *N. ureae* **(G,H)**, *Nitrosospira multiformis* **(I,J)**. Panels are single representative measurements of reproducible results (*n* = 3). Note differences in scale of x-axis for traces of NO production during NH_3_-oxidation.

Measurement of NO or N_2_O production during oxidation of NH_3_ or NH_2_OH were compared among the 5 strains and revealed that all AOB produce measureable quantities of NO during active oxidation of NH_3_ (**Figures 2A,C,E,G,I**). Although each AOB had a unique and dynamic NO production profile, making comparative rate calculations impractical, all strains produced>50 nM NO (per 1 × 10^10^ total cells) prior to anoxia in the MR chamber. *N. europaea* produced the least amount of NO compared to the other strains during active oxidation and prior to anoxia (**Figure 2A**; Supplementary Table S1). As reported previously (Kozlowski et al., 2016c) *N. multiformis* began re-consuming NO once *ca.* 50% O_2_ was left in the MR-chamber (**Figure 2I**) and both *N. europaea* and *N. communis* re-consumed a small amount of NO following anoxia in the MR-chamber (**Figures 2A,C**). Interestingly, either immediately upon O_2_ depletion in the case of *Nitrosomonas* sp. Is79A3 (**Figure 2E**) or *ca.* 5 min. post-anoxia for *N. ureae*, these two strains released massive quantities of NO outside the limit for measurement by the ami-600 NO microsensor (**Figures 2E,G**). Unlike the other AOB strains, neither *Nitrosomonas* sp. Is79A3 nor *N. ureae* re-consumed NO during active NH_3_-oxidation or following anoxia in the MR-chamber.

Measurement of NO during active substrate oxidation has so far only been studied in pure cultures of *N. europaea* (Kester et al., 1997; Yu and Chandran, 2010; Yu et al., 2010) and *N. multiformis* (Kozlowski et al., 2016c), both of which have annotated *nirK*, *norB*, and *norY* genes (**Table 1**). It is known, however, that the thaumarchaeotal ammonia-oxidizers (AOA) also produce NO during NH_3_-oxidation (Martens-Habbena et al., 2015; Kozlowski et al., 2016c); however, they retain very tight control over its production and consumption (Kozlowski et al., 2016c). There are significant similarities in NO profiles of the AOA *Nitrososphaera viennensis* and the oligotrophic AOB of the present study in that once O_2_ was depleted in the MR chamber substantial quantities of NO were released (**Figures 2E,G**; Kozlowski et al., 2016c). This similarity between the AOA and the oligotrophic AOB, both lineages with a low *K*_m_ and high affinity for ammonium (Martens-Habbena et al., 2009; Stahl and de la Torre, 2012; Prosser et al., 2014), could be explained by a lack of NOR genes to combat high intracellular NO experienced during anoxia either due to release of NO directly from the NH_3_-oxidation pathway, in the case of AOA (Kozlowski et al., 2016c), or perhaps from ${\mathrm{NO}}_{2}^{-}$ reduction in the case of the AOB (Stein, 2011). Importantly, the N_2_O measured from *N. viennensis* following NH_3_-oxidation and over an extended period of anoxia was a result of NO release and abiotic media-dependent conversion to N_2_O (Kozlowski et al., 2016c). Also, in the nitrifier-denitrification pathway of *N. europaea*, it should be noted that NorB is required for ${\mathrm{NO}}_{2}^{-}$ reduction to N_2_O (Kozlowski et al., 2014). This suggests that the lack of annotated NOR precludes a complete nitrifier-denitrification pathway in ammonia-oxidizers.

**FIGURE 3:**
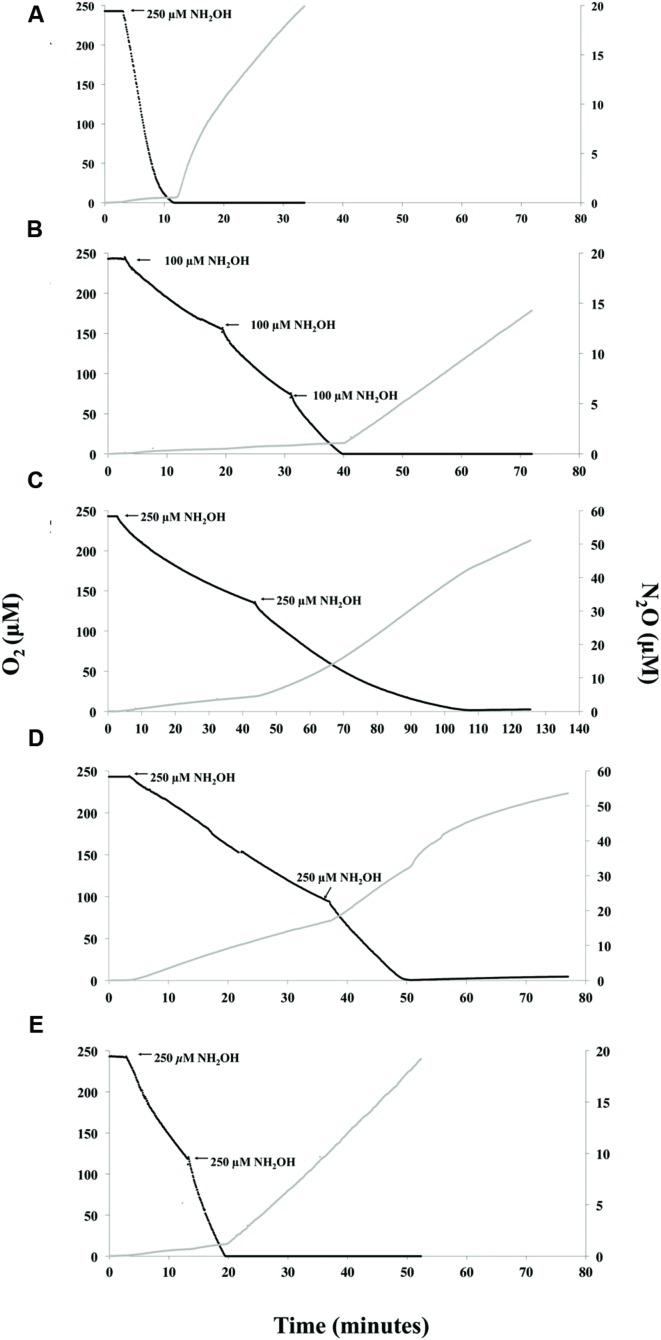
**Instantaneous measurement of O_2_ consumption and N_2_O production during oxidation of NH_2_OH.** *N. europaea* **(A)**, *N. communis* **(B)**, *Nitrosomonas* sp. Is79A3 **(C)**, *N. ureae* **(D)**, or *N. multiformis* **(E)**. Note the x-axis for *Nitrosomonas* sp. Is79A3 differs from the other traces. *Nitrosomonas* sp. Is79A3 and *N. ureae* have different y-axes for N_2_O production.

Following O_2_ depletion and in the presence of ${\mathrm{NO}}_{2}^{-}$ some AOB can perform nitrifier denitrification (Stein, 2011; Kozlowski et al., 2014). This was tested in the present study by measurement of N_2_O during active NH_3_- or NH_2_OH-oxidation and through a period of anoxia (**Figures 2** and **3**). It should be noted that the *K*_m_ for the copper-containing nitrite reductase, NirK, has not been tested for AOB and therefore it is not known whether *ca.* 162 or 243 μM ${\mathrm{NO}}_{2}^{-}$ following NH_3_ or NH_2_OH oxidation, respectively, in the chamber is at saturation for NirK.

Following NH_3_-oxidation, N_2_O was produced by all strains in the MR-chamber (**Figures 2B,D,F,H,J**). A greater delay of *ca.* 3 min in measureable N_2_O was seen from traces with both *N. communis* (**Figure 2D**) and *N. ureae* (**Figure 2H**). The lowest concentrations and slowest rates of N_2_O came from *N. communis* and *N. multiformis* (**Figures 2D,J**; Supplementary Table S1). *N. europaea* N_2_O production in the MR-chamber began immediately following O_2_-depletion and was produced at a rate of 0.47 μM N_2_O per 10^10^ cells^1^ per minute (**Figure 2B**; Supplementary Table S1). As with NO production, both *Nitrosomonas* sp. Is79A3 and *N. ureae* had similar N_2_O traces with similarly fast rates for N_2_O production following anoxia (**Figures 2F,H**; Supplementary Table S1).

With NH_2_OH as substrate, the majority of N_2_O in the MR-chamber from *N. europaea* (**Figure 3A**), *N. communis* (**Figure 3B**), and *N. multiformis* (**Figure 3E**) was produced in a linear fashion directly following anoxia suggesting enzymatic reduction of available ${\mathrm{NO}}_{2}^{-}$ to N_2_O and thus nitrifier denitrification. However, in the case of both *Nitrosomonas* sp. Is79A3 (**Figure 3C**) and *N. ureae* (**Figure 3D**) the majority of N_2_O was measured during active NH_2_OH-oxidation with production in both traces slowing upon complete O_2_-depletion. Furthermore, the quantity of N_2_O measured from both *Nitrosomonas* sp. Is79A3 and *N. ureae* during active NH_2_OH-oxidation was much greater overall than that produced from any other AOB, suggesting a greater overall release of NO, or other reactive intermediates, during this process (Law et al., 2012) (**Figure 3**).

It is interesting that *N. communis*, the only AOB lacking NirK, had very weak non-linear N_2_O production from NH_3_, yet strong linear production when NH_2_OH was provided (**Figures 2D** and **3B**). The linearity of N_2_O formation with NH_2_OH as substrate suggests that there is an enzymatic pathway for N_2_O formation under anoxic conditions, but this pathway is not active when NH_3_ is provided as substrate. This observation provides insight into the function of unidentified enzymology that links direct NH_2_OH oxidation to N_2_O production in *N. communis* that requires further investigation. Similarly, an *N. europaea* NirK deficient mutant was also able to reduce ${\mathrm{NO}}_{2}^{-}$ to N_2_O (Cantera and Stein, 2007; Kozlowski et al., 2014), further supporting the presence of alternate, as yet unidentified, NIRs in AOB.

### Contribution of AOB to Abiotic N_2_O

The N_2_O profiles of both *Nitrosomonas* sp. Is79A3 and *N. ureae* post-anoxia (**Figures 2F,H**) are congruent with a rapid and abundant release of NO (**Figures 2E,G**) being abiotically reduced to N_2_O, a characteristic trait observed in the AOA *N. viennensis* (Kozlowski et al., 2016c). Also in support of an abiotic origin of N_2_O for both *Nitrosomonas* sp. Is79 and *N. ureae* in comparison to the other AOB strains (**Figure 3**) is the observation that the majority of N_2_O was measured during active oxidation of NH_2_OH. Accumulation of NH_2_OH can lead to NO and N_2_O production at the active site of the HAO (Hooper and Terry, 1979; Stein, 2011). A high enough concentration of NO will react with components of the HK medium to form N_2_O as well (Kozlowski et al., 2016c). Interestingly, the lack of NirK did not cause significant production of N_2_O during active NH_2_OH-oxidation by *N. communis*, as shown previously for NirK-deficient *N. europaea* (Cantera and Stein, 2007), suggesting a different configuration of the ammonia-oxidation pathway among AOB that lack NirK.

In previous control experiments the intermediate NH_2_OH reacted with heat-killed cell moieties of the AOA, *N. viennensis* EN76, to produce abiological N_2_O (Kozlowski et al., 2016c). In the present study, abiotic and heat-killed cell controls were performed to demonstrate if NH_2_OH could react with either media components or heat-killed cells to produce N_2_O in the absence of active cellular functioning (Supplementary Figure S1). NH_4_^+^-free HK medium + NaNO_2_ or with heat-killed AOB and addition of 250 μM NH_2_OH showed that medium + NaNO_2_ or medium with heat-killed *N. europaea*, *N. communis*, and *N. multiformis* + NH_2_OH did not facilitate significant measureable N_2_O (Supplementary Figure S1). However, heat-killed cells of both *Nitrosomonas* sp. Is79A3 and *N. ureae* both produced measureable N_2_O following addition of 250 μM NH_2_OH. The reactivity of cellular moieties with NH_2_OH is further evidence of similarities among these oligotrophic AOB and the AOA as heat-killed controls of *N. viennensis* cells showed similar reactivity with NH_2_OH in growth medium (Kozlowski et al., 2016c). Taken altogether, the data support that N_2_O is produced abiotically from *Nitrosomonas* sp. Is79 and *N. ureae* similarly to that of the AOA, *N. viennensis*, likely due to their massive release of NO at anoxia and also the reactivity of their cellular moieties with NH_2_OH and other medium constituents.

## Conclusion

The present study highlights many new findings in the comparative phylogeny and nitrogen oxide metabolism of betaproteobaterial AOB. First, the data support the previous study of *N. europaea* that a cytochrome *c*-dependent NOR is required for nitrifier denitrification activity (Kozlowski et al., 2014). Second, the release of NO by the two oligotrophic strains in Cluster 6 of the AOB likely contributes to abiotic N_2_O production (chemo-denitrification), especially under environmental conditions that facilitate NO or NH_2_OH release (Jones et al., 2015; Zhu-Barker et al., 2015). This observation is congruent with the physiology of the oligotrophic AOA that lack NOR (Kozlowski et al., 2016c). Third, this study showcases the utility of comparative physiological studies on pure cultures of ammonia-oxidizers to characterize the diversity of mechanisms for NOx production and ultimately for N_2_O release to the environment.

## Author Contributions

JK and LS conceived the project; JK designed and performed all experiments, KK performed a PhyloPhlAn analysis and created the phylogenetic tree; JK, KK, and LS analyzed the data, JK and LS wrote and edited the manuscript.

## Conflict of Interest Statement

The authors declare that the research was conducted in the absence of any commercial or financial relationships that could be construed as a potential conflict of interest.

## Footnotes

1 http://www.ncbi.nlm.nih.gov/genome/
