# Supplementary material for: Comparison of Nitrogen Oxide Metabolism among Diverse Ammonia-Oxidizing Bacteria
Source: Front Microbiol. 2016 Jul 12;7:1090. doi: 10.3389/fmicb.2016.01090 (PMC4940428; doi:10.3389/fmicb.2016.01090)
Supplement: Supplementary file 1 [file Data_Sheet_1.PDF]

Supplemental Materials

Comparison of Nitrogen Oxide Metabolism Among Diverse Ammonia-Oxidizing Bacteria

Jessica A. Kozlowski, K. Dimitri Kits, Lisa Y. Stein*

***Correspondence:** Lisa Y. Stein: lisa.stein@ualberta.ca

Supplemental Table S1. Maximum NO produced during NH_3-oxidation prior to anoxia and rate of N_2O produced following NH_3-oxidation in anoxia. Data represent averages of replicate experiments (n=3) with standard error in parentheses.

	**Max. NO produced during NH_3-ox. prior to anoxia (nM NO x 10^{10} cells $^{-1}$)**	**Rate of N_2O production during anoxia following NH_3-ox. (μM N_2O/ 10^{10} cells $^{-1}$ / min.)**
***Nitrosomonas europaea* ATCC 19718**	65.81 (3.67)	0.47 (0.08)
***Nitrosomonas communis* Nm2**	218.38 (10.01)	0.22 (0.09)
***Nitrosomonas* sp. Is79A3**	215.30 (7.12)	4.69 (0.84)
***Nitrosomonas ureae* Nm10**	146.92 (17.97)	4.35 (1.15)
***Nitrosospira multiformis* ATCC 25196**	92.15 (17.95)	0.09 (0.04)

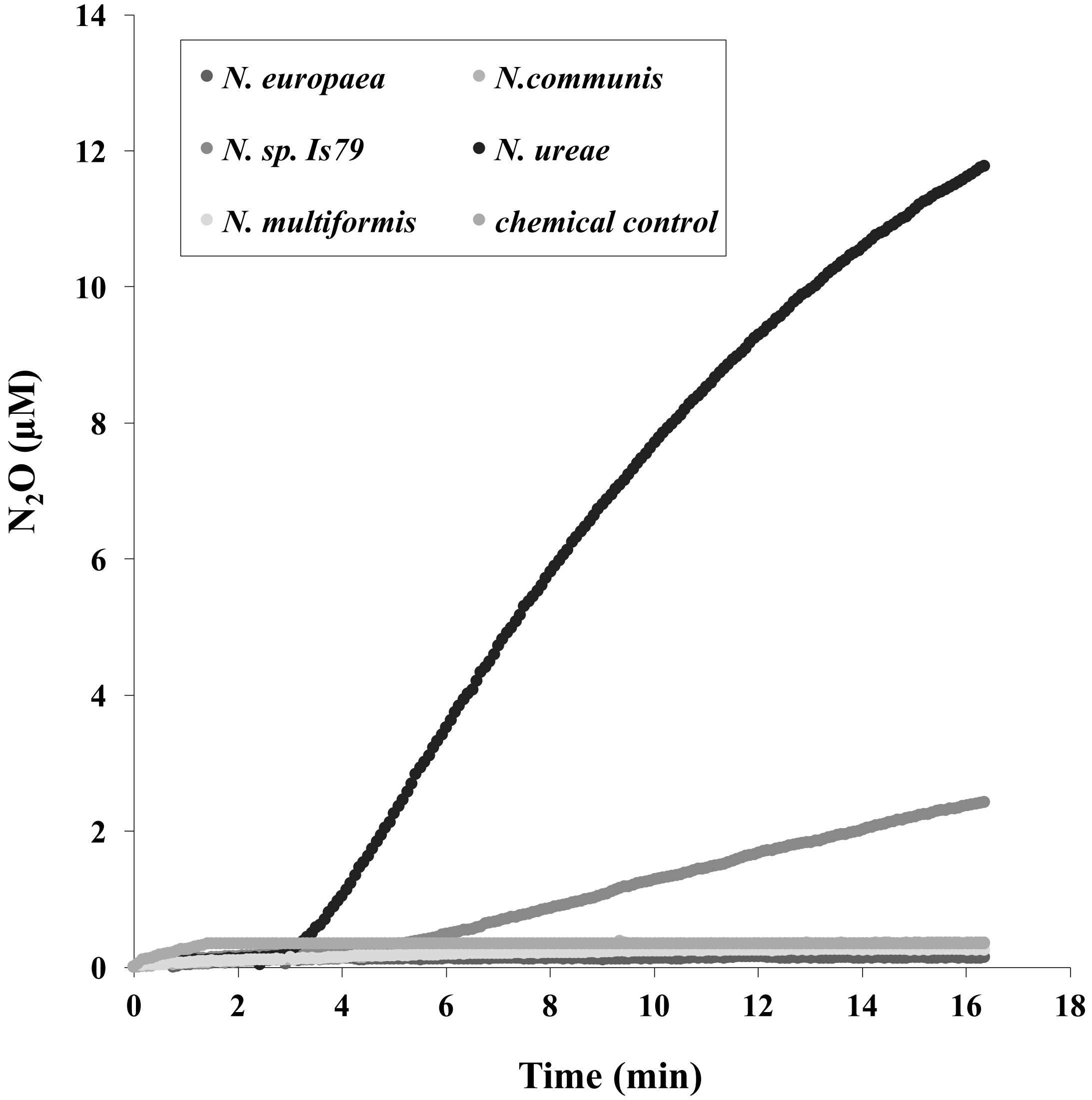

Supplemental Figure S1. Measurement of N_2O from either HK medium + 250 µM $NaNO_2^-$ or HK medium + killed cells with addition of 250 uM NH_2OH.
